# The Carbon Storage Regulator (Csr) System Exerts a Nutrient-Specific Control over Central Metabolism in *Escherichia coli* Strain Nissle 1917

**DOI:** 10.1371/journal.pone.0066386

**Published:** 2013-06-20

**Authors:** Olga Revelles, Pierre Millard, Jean-Philippe Nougayrède, Ulrich Dobrindt, Eric Oswald, Fabien Létisse, Jean-Charles Portais

**Affiliations:** 1 Laboratoire d’Ingénierie des Systèmes Biologiques et des Procédés, LISBP, Université de Toulouse, INSA, UPS, INP, Toulouse, France; 2 Laboratoire Ingénierie des Systèmes Biologiques et des Procédés, INRA UMR792, Toulouse, France; 3 UMR5504, CNRS, Toulouse, France; 4 USC1360, INRA, Toulouse, France; 5 U1043, Inserm, Toulouse, France; 6 UMR5282, CNRS, Toulouse, France; 7 Centre de Physiopathologie de Toulouse Purpan, Université de Toulouse, UPS, Toulouse, France; 8 Institute of Hygiene, University of Münster, Münster, Germany; University of Wisconsin-Milwaukee, United States of America

## Abstract

The role of the post-transcriptional carbon storage regulator (Csr) system in nutrient utilization and in the control of the central metabolism in *E. coli* reference commensal strain Nissle 1917 was investigated. Analysis of the growth capabilities of mutants altered for various components of the Csr system (*csrA*51, *csrB*, *csrC* and *csrD* mutations) showed that only the protein CsrA - the key component of the system - exerts a marked role in carbon nutrition. Attenuation of CsrA activity in the *csrA*51 mutant affects the growth efficiency on a broad range of physiologically relevant carbon sources, including compounds utilized by the Entner-Doudoroff (ED) pathway. Detailed investigations of the metabolomes and fluxomes of mutants and wild-type cells grown on carbon sources representative of glycolysis and of the ED pathway (glucose and gluconate, respectively), revealed significant re-adjusting of central carbon metabolism for both compounds in the *csrA*51 mutant. However, the metabolic re-adjusting observed on gluconate was strikingly different from that observed on glucose, indicating a nutrient-specific control of metabolism by the Csr system.

## Introduction

*Escherichia coli* is a normal inhabitant of the intestine and the predominant facultative anaerobe in the gastrointestinal tract of mammals [1]. The intestine is a highly complex and changing environment in which *E. coli* experiences constantly shifting growth conditions. Recent findings indicate that the ability to compete for carbon nutrition is a critical factor for gut colonization, and is part of the arsenal of strategies employed by pathogenic *E. coli* strains to outcompete the gut microbiotia [2], [3]. Colonization is mainly related to the utilization of sugars and sugar derivatives resulting from the degradation of mucus and of dietary fibers [4], [5]. Glycolytic pathways such as the Embden-Meyerhof-Parnas (EMP) and Entner-Doudoroff (ED) pathways play an important role in colonization [4], [6], [7], [8]. Persistence of *E. coli* in the gut is supported by less favorable substrates, including both sugars and non-sugar compounds such as the small organic acids resulting from the degradation of mucus by anaerobes of the microflora [4], [5]. The use of the latter compounds requires activation of gluconeogenic pathways, and efficient switching between glycolytic and gluconeogenic carbon sources is likely to be a major feature of successful adaptation to life in the intestine [9].

**Figure 1:**
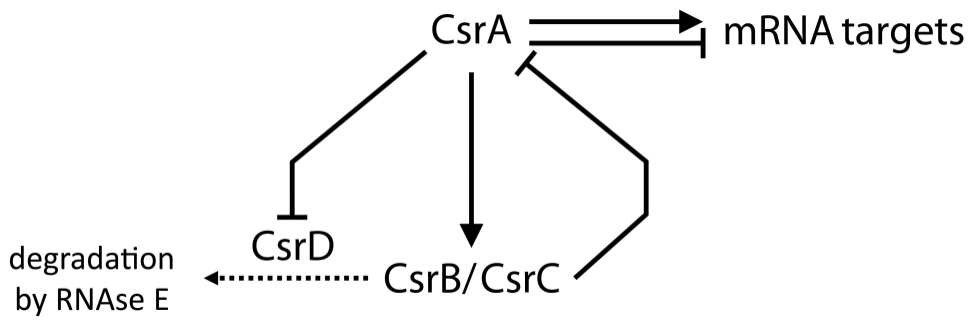
Regulatory network of the Csr system. Lines with an arrow denote positive control, and lines with a bar represent negative control.

To cope with the changing environment in the intestine, *E. coli* has developed a variety of adaptation mechanisms. Highly sophisticated global regulatory networks coordinate physiological and metabolic responses by controlling the functional expression of relevant sets of genes. The carbon storage regulator (Csr) system [10] is a post-transcriptional regulation system that controls a broad range of physiological adaptative mechanisms (including formation of biofilm, motility and virulence) and is a global regulator of central metabolism [11]. The Csr system has four molecular components (Figure 1). The main component is the post-transcriptional regulator CsrA, a protein that influences both translation and degradation of different mRNA targets [12]. CsrA is negatively regulated by two small non-coding RNAs, CsrB and CsrC, that antagonize CsrA activity by sequestering the protein [13], [14]. Lastly, the CsrD protein positively controls CsrA activity by driving the RNAs CsrB and CsrC to RNAse E degradation [15]. The molecular targets of the Csr system include a significant number of metabolic components. Indeed, CsrA is able to interact with most mRNAs of central metabolism enzymes [16], though the actual result of these interactions in terms of mRNA stability and translation is not known for most targets. Biochemical investigations have shown that CsrA exerts a positive control on several glycolytic enzymes (phosphoglucoisomerase, phosphofructokinase A, triose-phosphate isomerase, enolase and pyruvate kinase F) and on enzymes of acetate metabolism. In addition, it negatively controls gluconeogenic activities (fructose-1,6-biphosphatase, PEP synthase, PEP carboxykinase A) and glycogen biosynthesis (phosphoglucomutase, ADP-glucose pyrophosphorylase, glycogen synthase, glycogen phosphorylase) [17], [18], [19].

The global role of Csr in the control of metabolism suggests that this system could play a role in the adaptation of *E. coli* to changes in nutrient availability. Because it regulates glycolysis and gluconeogenesis in opposite ways, the Csr system is assumed to be a master switch between glycolytic and gluconeogenic metabolisms, and to increase competitiveness of *E coli* for the utilization of glycolytic carbon sources [9], [17]. Consistently, the CsrA protein is essential for growth on glucose – a glycolytic carbon source –, but not on a gluconeogenic compound like pyruvate [20]. However, despite advances in the molecular basis of Csr-associated regulation, the actual effects of Csr on the operation of *E. coli* metabolism – i.e. the actual metabolic phenotype - has not been fully investigated. The impact of Csr on its target metabolic pathways, in terms of metabolic pools and carbon fluxes, remains unknown. Indeed, the final metabolic phenotype may substantially differ from the direct action of Csr on its target enzymes. Metabolism is submitted to a highly complex regulation network which contains regulators that have effects opposite to that of Csr. The actual result of all the regulatory interactions is not fully predictable. In addition, the flux in a metabolic pathway is not correlated linearly with the activity of its enzymes, so that the effect of Csr on its target enzymes does not necessarily result in significant changes in pathway fluxes [21], [22]. Moreover, all metabolic pathways are tightly connected one to each other in the cell, and it is known that changes in some pathways can be compensated by the activation or inhibition of other pathways, in order to maintain metabolic homeostasis [22]. Finally, most molecular and biochemical studies on Csr were performed on *E. coli* cells grown on glucose, a carbon source which is not found in the colon, the main ecological niche of this bacterium. In contrast, very little is known regarding the role of Csr in the utilization and metabolism of physiologically-relevant carbon sources, which are often not metabolized by the same pathways as glucose and are not submitted to the same regulation networks. To fully understand the role of Csr in the adaptation to the gut environment, it is therefore necessary to investigate its role in the metabolism of physiologically relevant carbon sources.

In this work, we investigated the role of the Csr system in carbon nutrition and in the control of *E. coli* metabolism. First, we analyzed the capability of mutants altered for the various components of the Csr system to grow on a range of carbon sources that are found in the colon. Next, we studied the impact of Csr on the entire central carbon metabolism by analyzing the metabolomes and fluxomes of selected Csr mutants on two carbon sources, glucose and gluconate, which are metabolized via the EMP and ED pathways respectively. The identification of CsrA target enzymes and of the mechanisms by which their activity is controlled is out of the scope of this work. Because the deletion of *csrA* is lethal for growth on glucose and hence prevents investigation of the metabolic effects of CsrA on this carbon source - and potentially on other glycolytic compounds – the previously described attenuated CsrA protein CsrA_1-50_ was used in this study. The protein CsrA_1–50_ has a reduced RNA affinity and retains partial activity [20]. The *csrA51* mutant in which the native CsrA protein was replaced by the attenuated CsrA_1–50_ protein is viable on glucose [20], providing a valuable situation to investigate the role of the Csr system on a broad set of both glycolytic and gluconeogenic carbon sources. The study was carried out with the strain Nissle 1917, a non-pathogenic efficient colonizer of the gut [23], rather than with the classical domesticated *E. coli* such as K-12. This was done to ensure that all the physiological and metabolic mechanisms necessary for gut colonization and survival were in place and had not been affected by the prolonged lab use of K-12-like strains [24], [25]. Furthermore, Nissle 1917 belongs to the phylogenetic group B2, which is over-represented among *E. coli* strains persisting in the microbiota of humans from Europe, Australia, Japan and the USA [26].

## Materials and Methods

### Bacterial Strains and Genetic Methods

*E. coli* Nissle 1917 mutants were constructed using the allele replacement method described in [27]. Briefly, mutations were done by replacement of the target gene by an antibiotic-resistance cassette. The primers used for the constructions are listed in Table S1. The *csrB*::Cm^R^ locus was amplified by PCR using primers EH 12– EH 13 and strain RG1-B [28] as template, and the PCR product was used for *csrB* allelic replacement. For *csrC* mutagenesis, the *csrC*::Tc^R^ locus was amplified from TWMG1655 [14] with primers EH 10– EH 11. Strains containing multiple mutations were constructed by sequential allelic replacement; the first inserted cassette was removed with the FLP recombinase, followed by allelic replacement. All constructions were verified by PCR (Table S1). For complementation experiments, the *csrA* gene was amplified from chromosomal DNA and cloned into pGEM-T Easy (pCsrA). The resulting plasmid was transformed into *E. coli* Nissle Δ*csrA51* by electroporation. *E. coli* Nissle 1917 and its mutant derivatives are listed in Table 1.

**Table 1:** *Escherichia coli* strains used in this study.

Strain	Relevant genotype	Source
Wild-type	Wild-type *E. coli* strain Nissle 1917	Ardeypharm (Herdecke, Germany)
Δ*csrC*	*E. coli* Nissle 1917 *csrC*::Cm^R^	This study
Δ*csrB*	*E. coli* Nissle 1917 *csrB*::Tc^R^	This study
Δ*csrBC*	*E. coli* Nissle 1917 *csrC csrB*::Tc^R^	This study
Δ*csrD*	*E. coli* Nissle 1917 *csrD*::Cm^R^	This study
Δ*csrA*51	*E. coli* Nissle 1917 *csrA*51::Km^R^	This study
Δ*csrA*51 pCsrA	*E. coli* Nissle 1917 *csrA*51::Km^R^ pGEM-T *csrA*	This study

### Cultivation Conditions

Bacteria were grown at 37°C either in Lysogeny Broth (LB) containing no glucose, or in synthetic minimal medium supplemented with 15 mM of carbon source, as described elsewhere [29].

### Microreader Plate and Shake Flask Cultures

Detailed growth phenotyping of the strains was performed using real-time multiplex measurement of growth parameters based on fluorescent dyes sensitive to pH or oxygen and optical density, according to the method developed by [30]. Cells were grown in 48-well plates at 37°C in a volume of 500 µl of minimal medium, on a plate reader equipped with a shaker (Fluostar OPTIMA, BMG LABTECH, Ortenberg, Germany). Growth rates and durations of lag phase were determined as described in [30].

For in-depth metabolic characterization, cells were cultivated in 500 mL baffled flasks (37°C, 200 rpm) with 40 mL of medium. For ^13^C-labelling experiments, the unlabeled substrate (glucose or gluconate) was replaced by a mixture of 80% [1-^13^C]- and 20% [U-^13^C]- substrate (Eurisotop, Saint-Aubin, France). Inoculation was performed with cells pre-grown with the same labeled carbon sources and collected at mid-exponential growth phase, after centrifugation and washing with the same medium deprived of carbon source. All experiments were performed in three biological replicates.

### Glycogen Staining & Assay

To compare their glycogen content, the different strains were grown overnight at 37°C on LB plates containing 2% glucose and stained with iodine vapor [31]. Three independent experiments were carried out for each condition; the data shown are representative of one such experiment. To determine the rate of glycogen production upon exponential growth on glucose and on gluconate, glycogen content was quantified along the growth curve using the procedure described previously [32]. Briefly, glycogen was extracted and hydrolyzed to glucose monomers, which were quantified using an enzymatic method. All experiments were performed in triplicate.

### Motility Assay

A colony from a fresh LB plate was streaked on semi-solid LB plates (0.3% agar) and incubated upright at 37°C overnight. To determine the extent of bacterial motility, the diameter of the diffusion halo was measured after 24 h. Motility assays were determined in three independent experiments.

### Biofilm Assay

Cultures were grown statically overnight in 500 µl of LB, in a 48-well polystyrene microtiter plate. Biofilm formation was measured by discarding the medium, rinsing the wells (three times) with water, and staining bound cells with 20% crystal violet in methanol [33]. Absorbance was determined at 595 nm using a microtiter plate reader (Fluostar OPTIMA, BMG LABTECH). For each experiment, background staining was corrected by subtracting the crystal violet bound to an empty well.

### Growth Characterization

The growth rate (μ) was determined from log-linear regression of time-dependent changes in optical density at 600 nm (OD_600_), which was detected with a spectrophotometer (Genesys 6, Thermo Electron Corporation, MA, USA) with appropriate dilutions when needed. Extracellular fluxes were determined from the rates of disappearance (or appearance) of substrates and products, in the culture supernatants. The different compounds were quantified by HPLC (Agilent Series 1100, Agilent, CA, USA, on an Aminex HPX-87H column, Biorad, Hercules, CA, USA) as previously described [34]. To calculate specific biomass yields, correlation factors between cell dry weights and optical density (g_CDW_/OD_600_) were established for each strain and are given in Table S2.

### Sampling of Intracellular Metabolites for Metabolome Analysis

Samples were taken at the mid-exponential phase using the differential method [35]. Briefly, 120 µL of broth or filtered extracellular medium (Sartolon polyamid 0.2 µm, Sartorius, Goettingen, Germany) were plunged with 120 µL of fully ^13^C-labeled cellular extract (used as internal standard) in 5 mL of an ethanol/water (75/25) solution at 95°C, incubated for 2 minutes, cooled on ice and stored at −80°C. Three samples of broth and filtrate were taken and analyzed for each biological replicate.

### Sampling of Intracellular Metabolites for ^13^C-metabolic Flux Analysis

Sampling was performed at the mid-exponential phase in two steps; i) rapid quenching of metabolism followed by ii) metabolite extraction. For quenching, 120 µL of broth were rapidly sprayed into precooled centrifuge tubes maintained at −80°C and containing 500 µL of cold ethanol, homogenized using a vortex and centrifuged (12,000 g for 5 min at −20°C) with a Sigma 3–18K centrifuge (Sigma, Seelze, Germany). Metabolites were extracted by pouring 5 mL of an ethanol/water (75/25) solution at 95°C onto the cell pellets. After incubation for 2 min in closed tubes, the cellular extracts were cooled on ice and stored at −80°C. For each biological replicate, three metabolite samples were collected and analyzed.

### Preparation of Cellular Extracts and Ion-exchange-chromatography-MS(/MS) Analysis of Intracellular Metabolites

Cellular extracts were evaporated for 4 h (SC110A SpeedVac Plus, ThermoFisher, MA, USA). The remaining aqueous extracts were freeze-dried, resuspended in 200 µL of milliQ water and stored at −80°C. Intracellular metabolites were analyzed as previously described [36], [37]. Briefly, analysis was performed by high performance anion exchange chromatography (Dionex ICS 2000 system, CA, USA) coupled to a triple quadrupole QTrap 4000 (AB Sciex, CA, USA) mass spectrometer. All samples were analyzed in the negative mode by multiple reaction monitoring (MRM). The injection volume was 15 µL, originating from approximately 2 µg of biomass. For metabolomics experiments, the amounts of metabolites of glycolysis (G6P, F6P, FBP, PEP, 1,3-diPG and combined pools of 2- and 3-PG), ED and pentose phosphate (PP) pathways (6PG, R5P, S7P), TCA cycle (Mal, Suc, Cit, Fum, Aco), as well as nucleotides (ADP, AMP, ATP, cAMP, CDP, CMP, CTP, dADP, dATP, dCDP, dGTP, dTDP, dTTP, GDP, GMP, UDP, UDP-Glucose, UMP and UTP) were determined. To ensure highly accurate quantification, the isotope dilution mass spectrometry (IDMS) method was used [38]. For fluxomics experiments, the ^13^C-labeling patterns of central metabolites, including organic acids (Mal, Cit) and phosphorylated compounds (G6P, F6P, FBP, PEP, 6PG, R5P, S7P, combined pools of 2- and 3-PG) were determined as described in [37]. The labeling patterns (isotopologue distributions) were calculated from the isotopic clusters after correction for naturally occurring isotopes with IsoCor [39].

### NMR Analysis of Extracellular Medium

The labeling patterns of the main metabolic products – i.e. acetate and pyruvate - which accumulated during growth in the extracellular medium were measured by NMR spectroscopy [40], [41]. A total of 500 µl of broth was filtered and analyzed by quantitative ^1^H 1D-NMR at 298 K, using a 30° pulse and a relaxation delay of 20 s, with an Avance 500 MHz spectrometer (Bruker, Rheinstetten, Germany). The four acetate isotopomers and the eight pyruvate isotopomers were fully resolved and quantified.

### Fluxome Analysis

Fluxes were calculated using influx_s software [42], in which both mass balances and carbon atom transitions describing the biochemical reaction network were implemented. The metabolic network contained the main pathways of *E. coli* central metabolism: glycolysis (EMP), pentose phosphate pathway (PPP), Entner Doudoroff (ED) pathway, the tricarboxylic acid cycle (TCA), and anaplerotic reactions. The precursors required for biomass synthesis were estimated according to biomass composition [43] and measured growth rates. Intracellular fluxes were estimated from measurement of extracellular fluxes and from the ^13^C-labelling patterns of metabolites using appropriate mathematical models of glucose or gluconate metabolisms in *E. coli* [29]. Labeling data were collected from intracellular metabolites by IC-MS/MS and from metabolic end-products by 1D ^1^H NMR, as detailed above. The fluxes were normalized to the rate of substrate uptake, which was arbitrarily set at 100. To determine the confidence intervals of the calculated fluxes, we used a Monte-Carlo approach where 100 independent optimization runs were performed from datasets in which noise was added according to the standard deviations of the measurements. Isotopic data and metabolic fluxes for each independent biological replicate are given in Dataset S1.

### Calculation of ATP and Redox Balances

The adenylate energy charge (AEC) was calculated from the intracellular pools of adenosine mono-, di- and tri-phosphate according to [44]:
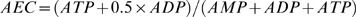



NADH, NADPH and FADH_2_: for each reduced cofactor, total production was calculated by summing the fluxes of all reactions which produced the reduced cofactor. The NADH that was synthesized in biosynthetic pathways, as well as NADPH requirements for anabolism, were estimated from biomass composition [43] and measured growth rates. The remaining NADPH was assumed to be converted into NADH via transhydrogenase activities.

ATP: production of ATP via substrate-level phosphorylation was calculated by adding all the fluxes measured for all ATP-producing reactions in carbon metabolism, and subtracting the fluxes of all ATP-consuming reactions. The ATP produced from NADH and FADH_2_ via oxidative phosphorylation was estimated assuming a P:O ratio of 1 for both redox cofactors [45]. ATP requirements were calculated by summing the requirements for anabolism, which were estimated from biomass composition, measured growth rates, and maintenance needs (including both growth associated and non-growth associated maintenance [45]).

### Statistical Analysis

Standard deviations of metabolic fluxes and intracellular pool sizes were determined from three biological replicates. Statistical differences in metabolite concentrations between the wild-type and mutant strains were calculated using Student’s paired *t*-tests with two tailed distributions.

## Results

### Construction and Phenotypic Characterization of Nissle 1917 Csr Mutants

To analyze the role of the different components of the Csr system, we first constructed null mutations in the *csrB, csrC, csrBC* (double mutant) and *csrD* genes of the *E. coli* strain Nissle 1917 by allelic exchange (Table 1, primers used in that study are listed in Table S1). For the construction of the double mutant Δ*csrBC*, a first single mutant Δ*csrB* was constructed (see Materials and Methods). Further, the resistant cassette was removed using FLP recombinase, followed by allelic replacement of the gene *csrC* with a chloramphenicol resistant gene. Consistent with the fact that the *csrA* gene is essential for growth on LB and glycolytic carbon sources [20], all attempts to generate a full *csrA* deletion mutant on glucose failed. A mutant of the laboratory *E. coli* strain K-12 MG1655 encoding a truncated CsrA protein was shown to be viable on glycolytic compounds, but was significantly impaired for processes controlled by CsrA [20]. A similar mutant (Δ*csrA*51) was successfully generated in strain Nissle 1917 to analyze the effects of attenuated CsrA activity. Mutants of the Csr system are expected to be altered for glycogen storage, biofilm formation and motility [10], [46], [47]. The different mutants obtained in this work were therefore phenotyped against these criteria. The glycogen content of the Csr mutants was measured by iodine staining. The Δ*csrB,* Δ*csrC,* Δ*csrBC* and Δ*csrD* mutants accumulated amounts of glycogen similar or slightly lower than the wild-type strain. In contrast, the Δ*csrA*51 mutant accumulated substantially more glycogen than all other strains (Table 2). Biofilm formation was estimated by measuring the crystal violet staining of the cells attached to the microtiter plate wells. The Δ*csrA*51 mutant formed dense biofilms while all other strains showed the same density as the wild-type. Growth measurements on semi-solid LB agar plates revealed that cell motility was reduced for both the Δ*csrA*51 and Δ*csrD* mutants but not for the other mutants. Thus the phenotype of the Nissle 1917 Δ*csrA*51 mutant was in close agreement with the phenotypes described for the laboratory strain MG1655, and those expected for the other Csr mutants.

**Table 2:** Phenotypic characterization of Nissle 1917 and its isogenic Csr mutants.

Strain	Glycogen	Motility	Biofilm
wild-type	++	++	+
Δ*csrA*51	++++	+	+++
Δ*csrB*	+	++	+
Δ*csrC*	+	+++	+
Δ*csrBC*	+	+++	++
Δ*csrD*	++	+	+

Each experiment was performed in three independent biological replicates. The data shown here are semi-quantitative.

### Metabolic Capabilities of *E. coli* Nissle 1917 and its Csr Mutants on a Variety of Carbon Substrates

**Figure 2:**
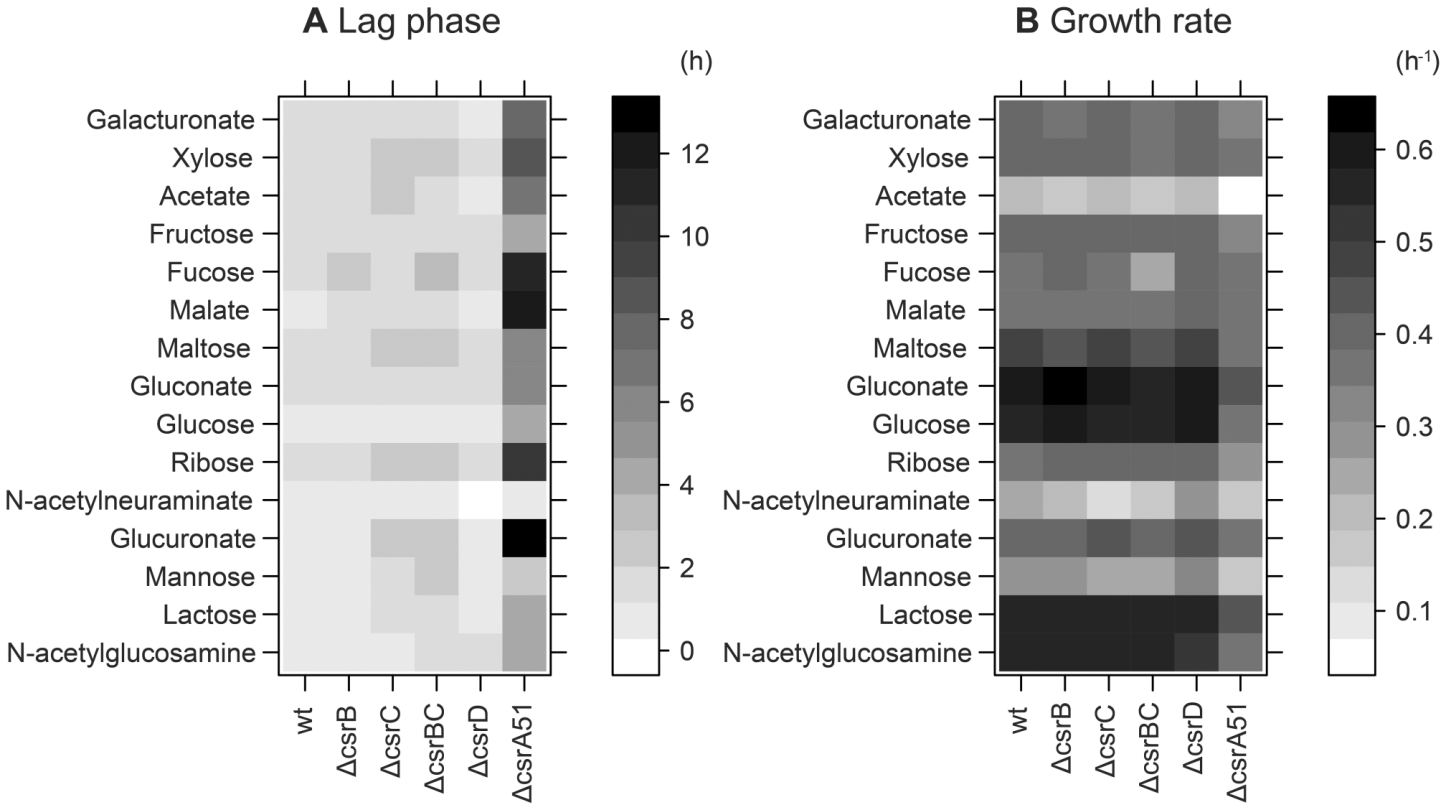
Growth phenotypes of *E. coli* Nissle 1917 and its Csr mutants. Heat maps representing the lag times (A) and growth rates (B) upon growth on 15 carbon sources representative of the gut environment. Rows represent different carbon sources and columns represent strains. In panel A, the gray scale indicates long (darker) to short (lighter) lag phase. In panel B, the gray scale indicates high (darker) to low (lighter) growth rate (B).

To determine the metabolic capabilities of *E. coli* wild-type strain Nissle 1917 and its Csr mutants, a large-scale physiological analysis was performed in which growth phenotypes were monitored on 15 carbon sources found in the gastrointestinal tract [4], [8] (Figure 2). Detailed information on growth was obtained using a multiplex monitoring system which allows the simultaneous follow-up of growth, pH and oxygen profiles in plates [30].

The growth phenotypes of the Δ*csrB,*Δ*csrC,*Δ*csrBC* and Δ*csrD* mutants were very similar to that of the wild-type strain (Figure 2). In contrast, the growth of the Δ*csrA*51 mutant was significantly altered. This mutant showed longer lag phases for all carbon sources tested (Figure 2A). The growth rates were also altered in a compound-dependent manner (Figure 2B). As expected, growth of the Δ*csrA*51 mutant was significantly reduced on most carbon sources utilized via the EMP pathway (glucose, mannose, lactose, N-acetyl-glucosamine or maltose), although no significant difference was observed for fructose and fucose. The latter two carbon sources enter glycolysis after the phosphofructokinase step (as fructose-1,6-bisphosphate and di-hydroxyacetone-1-phosphate, respectively), so that the activation of the latter enzyme by CsrA is not needed to utilize these compounds. In addition, the inhibition of fructose-1,6-biphosphatase by CsrA [17] would prevent block neoglucogenesis, which is an absolute requirement to fulfill the anabolic demands in hexoses-phosphates and pentoses-phosphates on both compounds. In agreement with the positive control exerted by CsrA on acetyl-CoA synthetase [19], the Δ*csrA*51 mutant grew poorly on acetate.

Furthermore, the gastrointestinal tract contains compounds that are used by the Entner-Doudoroff (ED) – i.e. gluconate, glucuronate - and Pentose-Phosphate (PP) – i.e. xylose, ribose - pathways. Although all strains grew similarly on xylose, growth on ribose was reduced by 50% in the Δ*csrA*51 mutant. This was unexpected as the Csr system is not assumed to control the PP pathway – at least in the K-12 strain MG1655 [48]. The role of the Csr system in the use of ED-utilized compounds has not been investigated so far. Our data show that the growth of the Δ*csrA*51 mutant on compounds degraded via this pathway was impaired. The growth rate was reduced by 10% and 20% on galacturonate and glucuronate, respectively, and by 30% on gluconate, compared to the wild-type strain. This indicates that the Csr system is involved in the utilization of the three compounds degraded by the ED pathway.

### Growth of *E. coli* Nissle and its Csr Mutants on Glucose and Gluconate

Physiological analysis of the Δ*csrBC* and Δ*csrA*51 mutants was performed upon growth on glucose and gluconate, as valuable representatives of nutrients utilized via the EMP and ED pathways (Table 3). As control, the Δ*csrA*51 mutant was complemented with a plasmid carrying the intact *csrA* gene (pCsrA). The complemented mutant displayed physiological parameters similar to those of the wild-type strain (Table 3).

**Table 3:** Growth parameters of Nissle 1917 and Csr mutants with glucose and gluconate as carbon source.

Substrate	Strain	*µ (h^−1^)*	*q_S_ (mmol.g_DW_^−1^.h^−1^)*	*v*_Ac_ *(mmol.g_DW_^−1^.h^−1^)*	Y*_Ac_* (%)	*v_Pyr_ (mmol.g_DW_^−1^.h^−1^)*	Y*_Pyr_* (%)
Glucose	wild-type	0.79±0.02	12.5±1.0	6.3±0.1	50±2	n.d.^a^	n.d.
	Δ*csr*BC	0.75±0.03	12.0±0.3	5.2±0.2	43±7	n.d.	n.d.
	Δ*csr*A51	0.63±0.02	10.8±0.7	7.2±0.1	67±5	n.d.	n.d.
	Δ*csr*A51 pCsrA	0.72±0.02	12.9±0.5	6.1±0.1	47±5	n.d.	n.d.
Gluconate	wild-type	0.74±0.02	20.0±1.7	10.4±0.1	52±3	3.2±0.1	16±2
	Δ*csr*BC	0.76±0.01	20.4±0.9	11.0±0.1	54±7	2.8±0.1	14±1
	Δ*csr*A51	0.57±0.01	12.6±1.3	6.3±0.1	50±5	0.4±0.3	3±1
	Δ*csr*A51 pCsrA	0.74±0.04	19.8±2.0	9.3±0.1	47±3	4.5±0.1	23±1

μ, specific growth rate; *q_S_*, carbon source uptake rate; *v_Ac_*, acetate production rate; Y*_Ac_*, acetate production yield ( = *v_Ac_*/*q_S_*); *v_Pyr_*, pyruvate production rate; Y*_Pyr_*, pyruvate production yield ( = *v_Pyr_*/*q_S_*). Values represent the mean ± standard deviation of three independent biological replicates.

a n.d.: not detected.

The wild-type strain Nissle 1917 grew on glucose at a rate of 0.79 h^−1^ with a glucose uptake rate of 12.5 mmol.g^−1^.h^−1^. The growth parameters of the Δ*csrBC* double mutant were very similar to those of the wild-type strain, despite the putative inability of this mutant to regulate free CsrA concentration due to the absence of the two regulatory small RNAs. In agreement with the data reported above, the physiology of the mutant Δ*csrA*51 was significantly altered, showing a longer lag phase, lower growth rate and lower substrate uptake compared to its parental strain. The conversion of glucose into acetate in the Δ*csrA*51 mutant was significantly higher than in the wild-type strain (67% vs. 50%), indicating less efficient use of the sugar.

Because the growth of the Δ*csrA*51 mutant was impaired on gluconate (Figure 2), and due to the importance of this carbon source in colon colonization, we investigated the metabolome of *E. coli* Nissle 1917 and its *csr* mutants upon growth on gluconate. The wild-type strain grew on gluconate at a rate similar to that observed on glucose (0.74 h^−1^ vs. 0.79 h^−1^), although much more gluconate than glucose (20.0 vs. 12.5 mmol.g^−1^.h^−1^) was needed to achieve the same growth rate, indicating different metabolic efficiencies. Gluconate was converted into two by-products, pyruvate and acetate, with molar yields of 0.16 and 0.52, respectively. Like on glucose, the growth of the Δ*csrBC* mutant did not significantly differ from that of the wild-type strain, whereas the mutant Δ*csrA*51 showed marked differences (Table 3): (i) the growth rate of the Δ*csrA*51 mutant was 20% lower than that of the two other strains, (ii) the lower growth rate was correlated with a 40% reduction in gluconate consumption, (iii) acetate production rate was reduced but acetate yield remained unchanged, and (iv) the production of pyruvate was drastically reduced and was almost negligible in the Δ*csrA*51 mutant.

### Metabolic Profiles of *E. coli* Strains Upon Growth on Glucose

To increase our understanding of the role of the Csr system in glucose metabolism, the metabolomes of the Δ*csrA*51 and Δ*cs*r*BC* mutants were compared to those of the wild-type strain. Absolute quantification of central and energetic metabolites was obtained from cells growing exponentially in minimal medium with glucose as the sole source of carbon. The results of the most representative metabolites are listed in table 4, while more details are given in Table S3. The metabolite content of the Δ*cs*r*BC* mutant generally did not significantly differ from that of the wild-type strain. In contrast, the mutant with a truncated CsrA protein underwent a dramatic increase in the levels of metabolites from the upper part of the EMP and PP pathways. The levels of F6P, 6PG and S7P, were two to three times higher than in the wild-type. Interestingly, a significant drop in the intracellular levels of FBP was observed in the Δ*csrA*51 strain, indicating a reduction in phosphofructokinase activity, a known target of CsrA. The level of M6P in the Δ*csrA*51 mutant was twice as high as in the wild-type. No changes were detected in metabolites of the lower part of glycolysis, such as 2- and 3-PG and PEP. Surprisingly, the pools of metabolites related to the TCA cycle (Fum, Mal and Suc) were lower than in the wild-type.

**Table 4:** Metabolite concentrations (µmol/g_CDW_) in the wild-type *E. coli* Nissle 1917, Δ*csrBC* and Δ*csrA*51 mutants.

	Glucose			Gluconate		
Metabolite	wild-type	*ΔcsrBC*	*ΔcsrA*51	wild-type	*ΔcsrBC*	*ΔcsrA*51
1,3-diPG^a^	2.28±1.06	3.89±3.76	1.90±0.15	4.45±0.56	3.30±3.00	3.68±0.73
2/3-PG	1.42±0.11	1.67±0.52	1.12±0.23	4.20±0.33	4.66±0.45	2.66±0.20
6PG	0.38±0.09	0.38±0.07	0.77±0.20	3.59±0.21	2.33±0.55	0.65±0.79
ADP	0.71±0.06	0.82±0.25	0.91±0.27	0.95±0.28	1.01±0.35	0.80±0.19
AMP	0.16±0.03	0.11±0.09	0.21±0.06	0.20±0.05	0.40±0.10	0.17±0.02
ATP	3.50±0.29	3.21±0.03	2.86±0.48	3.51±0.15	4.08±0.15	2.95±0.24
cAMP	0.01±0.02	0.00±0.02	0.01±0.01	0.01±0.01	0.01±0.01	0.01±0.03
Cit	1.01±0.90	0.81±0.33	0.40±0.14	0.27±0.10	0.35±0.25	0.57±1.05
F1P	0.03±0.01	0.04±0.01	0.02±0.01	0.19±0.11	0.21±0.05	0.14±0.05
F6P	0.44±0.03	0.41±0.03	0.90±0.08	0.26±0.02	0.19±0.03	0.19±0.01
FBP	2.01±0.55	2.30±0.12	1.44±0.32	2.95±0.47	2.51±0.14	1.82±0.09
Fum	0.59±0.34	0.56±0.16	0.44±0.09	0.75±0.26	0.72±0.10	0.63±0.15
G6P	1.78±0.19	1.93±0.61	2.00±0.40	0.97±0.70	0.48±0.05	0.39±0.05
M6P	0.26±0.04	0.26±0.04	0.56±0.17	0.05±0.03	0.12±0.01	0.15±0.01
Mal	1.82±0.45	0.93±0.30	0.95±0.30	1.64±0.57	2.29±0.29	2.35±0.67
PEP	0.19±0.05	0.21±0.08	0.21±0.05	0.49±0.05	0.54±0.12	0.49±0.11
R1P	0.02±0.01	0.01±0.001	0.01±0.01	7.81±0.81	42.2±9.2	17.9±4.3
R5P	0.75±0.06	0.73±0.28	0.71±0.15	1.93±0.45	2.60±0.16	3.38±0.41
S7P^a^	4.63±0.31	4.17±1.19	12.8±1.5	19.9±1.5	27.8±2.5	52.9±2.7
Suc	4.44±0.27	5.34±1.52	1.98±0.46	3.34±0.38	5.58±0.85	6.69±0.56
UDP-Glucose	4.6 5±0.14	4.86±0.47	5.20±0.47	2.86±0.31	3.79±0.57	2.14±0.56

Values represent the mean ± standard deviation of three independent biological replicates.

a For these metabolites only the ^12^C/^13^C ratio is given. This ratio represents the concentration of the metabolite in samples relative to the IDMS standard, which added at the same amounts in all samples.

The adenylate energy charge (AEC) was measured from the ATP, ADP and AMP contents listed in Table S4. No significant differences were observed between the wild type and the mutants, providing evidence that all strains were able to maintain a stable energetic status.

### Metabolic Flux Responses in the Csr Mutants Upon Growth on Glucose

To better understand the effect of the Csr system on central metabolism, the distribution of metabolic fluxes was analyzed in the wild-type strains and in Δ*csrBC* and Δ*csrA*51 mutants. For this purpose, steady-state ^13^C-labeling experiments were carried out in which the strains were fed with a mixture of 80% 1-^13^C and 20% U-^13^C-glucose as sole carbon source.

We first wondered about the flux toward glycogen production as this is assumed to be responsible for the growth deficiency of a Δ*csrA*51 partial deletion mutant [20]. The rate of glycogen production and glycogen accumulation was calculated during the exponential phase of growth on glucose. The flux values obtained for glycogen synthesis in both the wild type and in the Δ*csrA*51 mutant were in the range of µmoles.g^−1^.h^−1^ (Table 5). This is 3 to 4 orders of magnitude lower than the rates of substrate uptake (in the range of 10 to 20 mmol.g^−1^.h^−1^) or the fluxes observed in the main metabolic pathways. Thus, the flux of carbon diverted towards glycogen formation in the Δ*csrA*51 mutant is much too low to significantly alter the overall carbon flux, and is likely not the main reason for the flux redistribution observed in exponentially growing cells for this mutant.

**Table 5:** Glycogen contents and glycogen production rates in exponential growth phase on glucose and gluconate.

	Glycogen content (µmolGlc.g_CDW_^−1^)		Glycogen production rate (mmol.g_CDW_^−1^.h^−1^)	
Strains	Glucose	Gluconate	Glucose	Gluconate
wild-type	3.9±0.4	4.8±0.4	0.003±0.001	0.001±0.001
Δ*csrA*51	47.2±0.1	8.5±1.8	0.057±0.014	0.007±0.001

For glycogen content, µmolGlc denotes the amount of glucose monomers. Values represent the mean ± standard deviation of three independent biological replicates.

**Figure 3:**
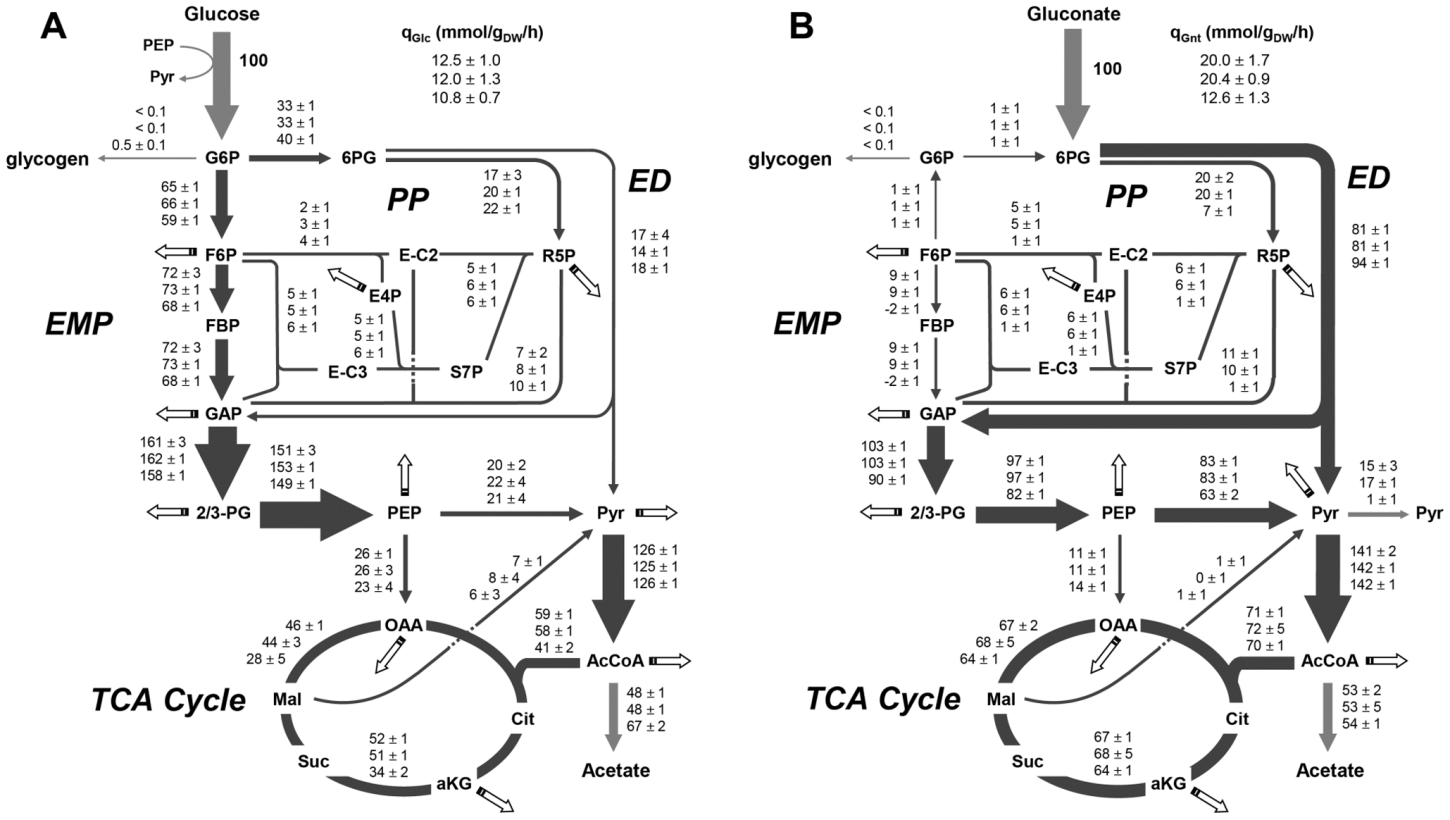
Metabolic flux distributions upon growth on glucose (A) and gluconate (B) as carbon source. Values from top to bottom: wild-type strain, Δ*csrBC* and Δ*csrA*51. Flux values are normalized to the specific glucose uptake rate of each strain, which was arbitrarily given the value of 100. Values represent the mean ± standard deviation of three independent biological replicates for each strain. Arrow thicknesses represent the flux values in the wild-type strain. Box arrows represent fluxes towards biomass synthesis.

The flux data obtained for the wild-type Nissle 1917 stain (Figure 3A) showed that glucose is predominantly catabolized via the EMP pathway. The fraction of glucose catabolized via the PP pathway (16%) was similar to that observed for other strain, meanwhile the contribution of the ED pathway (17%) to glucose catabolism was significant compared to other strains [29]. The flux distribution in the Δ*csrBC* mutant (Figure 3A) did not differ significantly from that in the wild-type strain, although the split ratio between the PP and the ED pathway was slightly higher. The flux distribution in the Δ*csrA*51 mutant showed more differences. The relative flux through the EMP in the mutant was lower than in the wild-type. As mentioned above, this was not due to increased flux towards glycogen synthesis, but to a redirection of the carbon flow towards the PP pathway. Indeed, the EMP/PP split ratio was decreased from 2 in the wild-type to 1.5 in the Δ*csrA*51 mutant. The major differences between the Δ*csrA*51 mutant and the wild-type strain were strikingly lower fluxes via the TCA cycle for the mutant than in the wild-type, while no differences were observed for anaplerotic fluxes. The fluxes carried out by citrate synthase and succinate dehydrogenase were 30% and almost 40% lower, respectively. The decrease in the TCA cycle in the Δ*csrA*51 mutant was accompanied by a 40% increase in acetate production. These differences were shown to be statistically significant (*p*<0.01) by the sensitivity analysis applied to each flux data set, and reproducible in three biological replicates.

**Figure 4:**
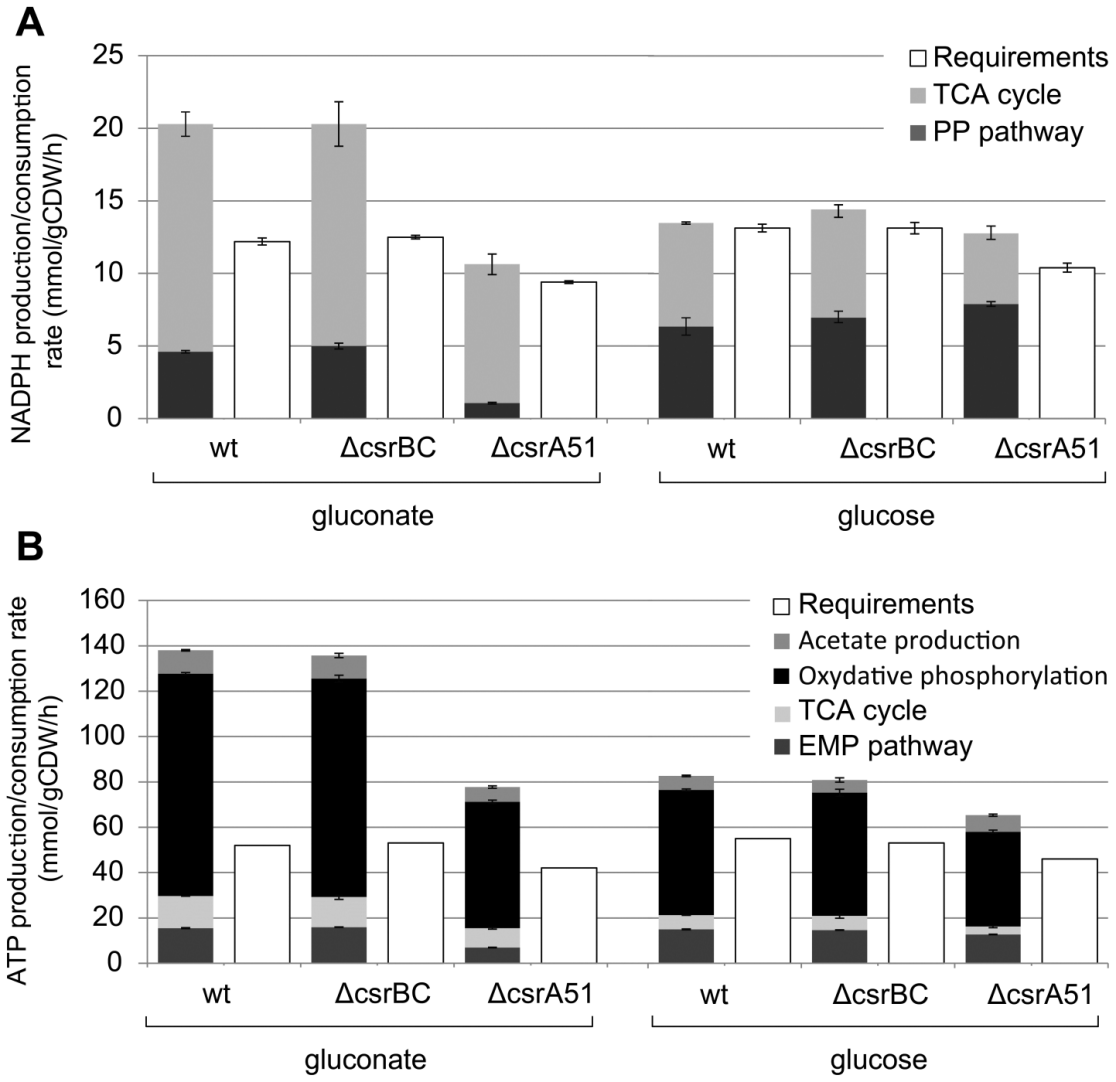
NADPH (A) and ATP (B) balances in upon growth on glucose and gluconate. Productions of cofactors were calculated from carbon fluxes. The requirements were calculated on a theoretical basis from biomass composition and growth rates.

To further evaluate the impact of the Csr mutations on *E. coli* Nissle 1917 metabolism, the production of high-energy molecules NADPH and ATP was calculated from the ^13^C flux data. In the wild-type strain and the Δ*csrBC* mutant, the PP pathway and the TCA cycle contributed almost equally to the production of NADPH. The contribution of the TCA cycle was significantly (around 30%) lower in the Δ*csrA*51 mutant and was compensated for by the PP pathway. In all strains, the production of NADPH was sufficient to fulfill the anabolic needs (Figure 4). To establish the ATP balance, the ATP requirements for growth and maintenance energy were calculated [45], and the ATP production was calculated in the different strains. In all strains, total ATP production was higher than the predicted requirements (Figure 4). The absolute production of ATP in the Δ*csrA*51 mutant was 20% lower than in the two other strains, which was consistent with the lower growth rate. Substrate-level phosphorylation and oxidative phosphorylation represented 30% and 70% of total ATP production, respectively. This percentage remained remarkably constant in all strains, including in the Δ*csrA*51 mutant.

### Metabolic Profiles of Cells Grown on Gluconate

Like on glucose, on gluconate, there were no significant differences between the metabolome of Δ*csrBC* and the wild-type strain. In contrast, significant differences (*p*<0.01) were observed in the Δ*csrA*51 mutant in the upper part of carbohydrate metabolism (Table 4 & Table S3). A drastic 5-fold reduction was observed in the intracellular level of 6PG, the first intermediary of gluconate utilization. Conversely, the intracellular levels of the PP pathway metabolites R5P, R1P and S7P, were 2 to 3-fold higher in the Δ*csrA*51 strain. The EMP metabolites in this mutant were 2 times lower than in the wild-type, including F6P, FBP and 2- and 3-PG. The levels of the TCA cycle metabolites Suc and Cit increased significantly, while the concentration of Fum remained almost stable. The level of M6P increased dramatically. As observed on glucose, the AEC was similar in all the strains analyzed, indicating a stable energetic status.

### Metabolic Flux Responses in Csr Mutants on Growth on Gluconate

The metabolic flux distribution in cells grown on gluconate was determined from steady-state ^13^C-labeling experiments (Figure 3B) with ^13^C- a mixture of 80% [1-^13^C]- and 20% [U-^13^C]-gluconate as sole carbon source, similarly to the experiments carried out with labeled glucose. Here again, we found that the flux from glycolysis to glycogen was negligible in all three strains growing on gluconate. In the wild-type *E. coli* Nissle 1917, gluconate was mainly used via the ED pathway (80% of gluconate uptake). A significant proportion was also used via the PP pathway, and the PP/ED flux ratio was 20∶80. A small proportion of hexose 6-phosphate was also recycled in the ED and PP pathways. The conversion of GAP into pyruvate in the lower part of glycolysis was much lower than observed on glucose (80% and 130% of substrate uptake, respectively). However, on gluconate, pyruvate is also generated via the ED pathway. The total flux of pyruvate formation by the two EMP & ED glycolytic pathways was higher on gluconate than on glucose (164% vs. 144% of substrate uptake, respectively). Flux data indicated that 10% of the pyruvate produced was released into the medium as a fermentation by-product of gluconate, and that 86% was converted into acetyl-CoA via pyruvate dehydrogenase. The TCA cycle used 51% of acetyl-CoA, while 38% was released as acetate. From flux data and the data listed in Table 3, it was possible to calculate that 35% of the gluconate molecules entering the cells were fully oxidized to CO_2_ (via the combined action of the glycolytic pathways and the TCA cycle), while 34% were converted into by-products (pyruvate & acetate). This is significantly different from what happened on glucose, where 25% of the molecules were fully oxidized by the combined action of glycolytic pathways+the TCA cycle, and 25% were released as a by-product (acetate).

Here again, the relative flux distribution in the Δ*csrBC* mutant was no different from that in the wild-type strain. Not surprisingly, significant differences were observed in the Δ*csrA*51 mutant. In this strain, the PP flux (i.e. flux through 6-phosphogluconate dehydrogenase) fell from 20% to 7% of the substrate uptake rate. While the ED flux increased from 81% in the wild-type to 94% in Δ*csrA*51, indicating almost pure ED metabolism. The formation of hexose 6-phosphate via the PP pathway stopped. Instead, hexose phosphates were obtained by recycling of the triose phosphate generated by the ED pathway, as shown by the negative flux in the series of reactions from F6P to GAP.

The flux in the lower part of glycolysis in the Δ*csrA*51 mutant was reduced compared to that in the wild-type strain. The reduced formation of pyruvate by lower glycolysis was compensated for by an increase in direct production of the latter compound via the ED pathway. Indeed, the total pyruvate flux was almost the same in the two strains (158% vs. 164% of substrate uptake, respectively). However, pyruvate utilization differed significantly in the two strains, since the excretion of pyruvate was almost abolished in the Δ*csrA*51 mutant. Unlike growth on glucose, the relative fluxes through the TCA cycle and acetate production were not altered in the Δ*csrA*51 mutant for growth on gluconate. These data showed that the reduced activity of the CsrA protein in this mutant did not affect the fraction of gluconate molecules that were fully oxidized to CO_2_ (34% vs. 35% in the wild-type strain).

The NADPH and ATP balances in the different strains were calculated for growth on gluconate. Compared to glucose metabolism, whatever the strain, the production of NADPH for growth on gluconate relied more on the TCA cycle than on the PP pathway. This phenomenon was amplified in the Δ*csrA*51 mutant, in which 90% of NADPH was produced in the TCA cycle, compared with 76% in the wild-type strain. In that respect, the effect of the *csrA*51 mutation was the reverse of the situation observed for growth on glucose, when the contribution of the TCA cycle decreased.

The ATP balance for growth on gluconate was determined in the wild-type, Δ*csrA*51 and Δ*csrBC* strains (Figure 4). As observed on glucose, total ATP production calculated from flux data was consistently higher than predicted requirements. The maximum amount of ATP produced on gluconate in the wild-type strain was significantly higher than on glucose (138 vs. 83 mmol.g^−1^). This is consistent with the fact that much more gluconate than glucose is consumed to obtain similar growth rates. As expected, there were no significant differences between the Δ*csrBC* mutant and the wild-type strain. In the Δ*csrA*51 mutant, ATP production was drastically reduced (77 mmol.g^−1^). Nevertheless, the respective contributions of substrate-level phosphorylation and oxidative phosphorylation to ATP production were constant in all three strains, as observed on glucose.

## Discussion

The Nissle 1917 strain belongs to the phylogenetic B2 group, which contains efficient gut colonizers. Consistently, this strain shows growth rates higher than the laboratory strains (K-12 strains) upon similar growth conditions. The higher rates of substrate uptake provide advantage in the gut environment since it decreases the availability of carbon nutrients for competing strains or organisms. The metabolic differences between the two types of strains can be detailed for cells grown on glucose, for which metabolome and fluxome data are available for both of them. In K-12 strains, glucose is predominantly catabolized via the EMP pathway, with 20% contribution of the PP pathway, while the ED pathway displays negligible activity [21], [29], [49]. In *E. coli* Nissle 1917, the flux in the PP pathway is similar to that in the K-12 strains, but a striking difference between the two strains is the significant contribution of the ED pathway (17%) to glucose catabolism in the B2 strain. The ED pathway plays a critical role in the implantation of *E. coli* in the colon because it allows the utilization of compounds – such as gluconate and other sugar acids utilized by this pathway - that are abundant therein [2], [4], [6], [7]. Hence it can be hypothesized that the activity of the ED pathway on glucose could be a competitive advantage for *E. coli* to switch rapidly from the utilisation of glucose to that of the ED-compounds. In addition, *E. coli* possesses a periplasmic glucose dehydrogenase (GDH) that converts glucose into gluconate, which is further used via the ED pathway. This oxidative glucose utilization process is not active in vitro because the cells do not produce pyrroloquinoline-quinone (PQQ), the cofactor of the periplasmic GDH. But PQQ is available in the gut, meaning that a part of glucose could be utilized by the process in situ [50]. Whatever the case, our data indicate that the regulation of the ED pathway in strain Nissle 1917 is different to that in K-12 strains. As a consequence of the operation of this pathway, the total flux through the two dehydrogenating pathways (PP+ED pathways) in the strain Nissle 1917 is significantly higher than that reported in K-12 strains (33% vs. 20%). This results in higher production of NADPH, which is required to fulfil biosynthetic needs in redox equivalents. Indeed, the amounts of NADPH produced within carbohydrate metabolism in strain Nissle 1917 are significantly higher than in K-12 strains. In the latter strain, two main pathways - i.e. the PP pathway, and TCA cycle - produce NADPH, although a slight contribution of the malic enzyme is sometimes reported [21], [49]. In the Nissle strain, three main processes contribute to NADPH: the PP pathway, the ED pathway and the TCA cycle, with a slight contribution of the malic enzyme. Due to the flux values in these processes, sufficient NADPH is produced within central carbon metabolism in the Nissle strain to fulfill all biosynthetic requirements. This is not the case in K-12 strains, in which additional contribution of a membrane-bounded transhydrogenase is required [49]. This transhydrogenase transfers redox equivalents from NADH to NADP^+^ at the expense of the transmembrane proton gradient, which means that part of the energy is consumed to produce sufficient NADPH in K-12 strains. Hence, significant differences in NADPH-producing mechanisms exist between the two types of strains, which might – at least partly – explain their differences in growth rates.

The present work provides also detailed insights into the metabolism of gluconate, which plays a major role in the colonization of the gut by *E. coli*. The data reported here for the wild-type Nissle strain are generally consistent with current knowledge of gluconate metabolism in *E. coli*, i.e. gluconate is majorly metabolized via the ED pathway. As observed upon growth on glucose, all biosynthetic requirements in NADPH are fulfilled from the operation of carbon pathways, without the need for additional transhydrogenase activity. This means that such metabolic capability of the Nissle strain is likely independent of the carbon source, and might be a specific trait of the strain. A striking feature of growth on gluconate is the significant production of pyruvate, which represents 15% of gluconate uptake, while the production of acetate is comparable to that observed on glucose (50% of gluconate uptake). Because the rate of gluconate uptake was very high and no oxygen limitation was observed, we hypothesized that pyruvate excretion on gluconate is due to overflow metabolism. Consistently, pyruvate production is abolished in the Δ*csrA*51 mutant which shows decreased gluconate uptake rates, and is not observed upon growth on glucose where the substrate uptake – hence, the intracellular rate of pyruvate synthesis – is significantly lower.The above observations indicate significant differences in the operation of central carbon metabolism between the Nissle 1917 strain and K-12 strains for two major carbon sources. Further investigations are needed to determine whether such differences are due to particular traits of the Nissle strain, or if they represent characteristics features of strains belonging to the phylogenetic B2 group.

The Csr system is thought to reinforce the competitiveness of *E. coli* in the use of carbon sources metabolized through the upper part of the EMP such as glucose [20], [51]. The data reported here reinforce and extend this role by showing that CsrA enhances the use of a wide range of carbon sources. The Csr system plays no role in nutrient selection (i.e. there was no alteration in Csr mutants in the spectrum of carbon sources used) but enhances growth efficiency. These effects were observed for compounds metabolized by different metabolic pathways. These results indicate that Csr either directly or indirectly controls a wider range of metabolic pathways than expected from its known target enzymes. Specifically, the work reported here demonstrates a role for Csr in the use of substrates metabolized via the ED and PP pathways. This is of particular physiological importance due to large number of such compounds in the colon and the reported role of the ED pathway in gut colonization [4], [7].

The significant re-adjusting of central carbon metabolism, resulting in both metabolome and fluxome changes - occurring in the Δ*csrA*51 mutant shows that CsrA indeed plays a significant role in the control of cellular metabolism. The metabolic effects depend on the nature of the carbon source, and striking differences were observed between the two carbon sources investigated, i.e. glucose and gluconate. The effects observed on glucose metabolism are generally consistent with the known role of the Csr system. Less efficient use of glucose in the Δ*csrA*51 mutant, with a reduced glycolytic flux, oxidative metabolism (reduced TCA cycle activity) and increased acetate production were all observed. These effects can be explained by the known targets of CsrA, since the positive control of glycolysis and the negative control of acetate metabolism triggers the carbon flow towards the TCA cycle. However, a direct control of the latter by CsrA, though not yet described, cannot be excluded. All these data suggest that CsrA plays a significant global role in the control of central metabolism on glucose by triggering carbon flow towards complete substrate oxidation (glycolysis+oxidative metabolism). These effects are likely to explain how the Csr system contributes to a higher metabolic efficiency on glucose.

The deletion of the gene encoding for CsrA in the laboratory *E. coli* strain MG1655 was reported to be lethal for growth on glucose due to a drastic diversion of the glycolytic flow towards glycogen synthesis [17], [18]. Accordingly, we could not generate a deletion mutant viable for growth on glucose in strain Nissle 1917. The Δ*csrA*51 mutant studied here accumulated significant amounts of glycogen during the stationary phase of growth, but the carbon flux towards glycogen synthesis measured during the exponential growth phase was much less than the glycolytic flux. Hence, the increased glycogen synthesis in this mutant is responsible for neither the decreased growth efficiency nor for the observed metabolic rearrangements. Because CsrA activity is only reduced but not suppressed in the *ΔcsrA*51 mutant, our results cannot exclude that in a CsrA-deletion mutant the effect on glycogen synthesis is much higher and can be responsible for the lethal phenotype on glucose. However, glycogen synthesis and its regulation are strain dependent [52], and actual effects in Nissle 1917 may differ from those in the MG1655 strain. It is worth noting that due to its evolution in the laboratory, MG1655 remains a poor colonizer compared to Nissle 1917. Furthermore, we showed in this work that the actual metabolic effect of CsrA is not only restricted to carbon sources that enter metabolism at the level of G6P, hence it cannot be explained by an increase in glycogen metabolism.

The detailed investigations of gluconate metabolism revealed surprising features. There is currently no biochemical evidence that Csr targets enzymes of the PP pathway, and only an indirect – as yet unidentified – action of CsrA on one of the ED enzyme (ED aldolase) has been proposed [53]. Due to the positive action of CsrA on ED aldolase and the absence of action on PP enzymes, a reduced contribution of the ED pathway to gluconate metabolism and a higher contribution of the PP pathway were expected in the Δ*csrA*51 mutant. However, the opposite effects were observed. Both the metabolome and fluxome data showed that gluconate was metabolized almost exclusively via the ED pathway, while the contribution of the PP pathway was strongly reduced. Contrary to the observations made on glucose, the relative fluxes in the TCA cycle and acetate metabolism in the mutant did not differ from that in the wild-type strain. Moreover, the wild-type strain released significant amounts of pyruvate as a by-product, but this process was almost absent in the Δ*csrA*51 mutant. The complete metabolisation of gluconate by the ED pathway is also accompanied by the concomitant inversion of the carbon flux in upper glycolysis, which operates in a gluconeogenic direction, resulting in the recycling of 6PG. Such recycling of triose phosphate has been already observed in ED-using species [54], [55]. All these observations show that i) the Csr system exerts significant and global control over metabolism during growth on gluconate, and ii) the re-adjusting of central metabolism in the Δ*csrA*51 mutant upon growth on gluconate differs significantly from that observed on glucose. The regulatory mechanisms by which the Csr system exerts global control over central metabolism upon growth on gluconate remain unclear. Trioses-phosphate recycling in the Δ*csrA*51 mutant is consistent with the known antagonistic effects of CsrA on glycolysis and gluconeogenesis, which is in favor of the latter process in the with Δ*csrA*51 mutant. It was recently reported that CsrA interacts with mRNAs transcribed from genes of the PP (*zwf*) and ED (*edd*) pathways [16]. These data suggests a potential role of Csr in the control of PP and ED enzymes, which could explain part of the metabolic re-adjusting observed in the Δ*csrA*51 mutant (“pure” ED metabolism, decreased pyruvate production). However, the effect of CsrA on PP and ED enzymes could not be clearly established [53], [56]. The molecular origin of the metabolic changes observed on gluconate is therefore unclear. In addition, the decreased acetate metabolism and decreased TCA cycle observed on glucose were not observed on gluconate, despite the action of Csr on these processes was expected to be the same. These means that flux regulatory mechanisms may strongly depend on the carbon source. The Csr system is embedded in a complex regulatory network. Thus, the global control exerted by Csr on *E. coli* metabolism is likely triggered through indirect mechanisms resulting from the interplay between the various regulation systems. To identify these regulatory mechanisms, further molecular investigations combining proteomics and transcriptomics should be performed, in light of the metabolome and fluxome data obtained here. Thus, a more detailed study should be done to identify CsrA enzyme targets, as well as, the molecular mechanism behind such regulation.

Whatever the regulatory mechanisms involved, this work shows that Csr is involved in the utilization of ED compounds and other physiologically-relevant carbon sources. Hence, CsrA appears as a key regulator for establishment and persistence of *E. coli* in the changing environment of the intestine. Because it influences the operation of *E. coli* metabolic network, the Csr system is also a potential target for biotechnologically-driven manipulations of the metabolism of this bacterium [56].

## Supporting Information

Table S1 List of primers used in this study.
(DOC)

Table S2 Conversion factors between cell dry weight and optical density (OD_600_) for the wild-type *E. coli* Nissle 1917, Δ*csr*BC and Δ*csrA*51 mutants.
(DOC)

Table S3 Metabolite concentrations in the wild-type *E. coli* Nissle 1917, Δ*csr*BC and Δ*csrA*51 mutants. Statistical differences between wild-type and Δ*csrA*51 mutant are given.
(DOC)

Table S4 Adenylate energy charge.
(DOC)

Dataset S1 Metabolic fluxes and isotopic data in the wild-type *E. coli* Nissle 1917, Δ*csr*BC and Δ*csrA*51 mutants.
(XLSX)
