# Supplementary material for: The Carbon Storage Regulator (Csr) System Exerts a Nutrient-Specific Control over Central Metabolism in Escherichia coli Strain Nissle 1917
Source: PLoS One. 2013 Jun 20;8(6):e66386. doi: 10.1371/journal.pone.0066386 (PMC3688793; doi:10.1371/journal.pone.0066386)
Supplement: Table S1 — List of primers used in this study. (DOC) [file pone.0066386.s001.doc]

**Table S1.** List of primers used in this study.

| **Gene Name** | **Primer Name** | **Primer Sequence** | **Application** | **Size of PCR Fragment (bp)** |
| --- | --- | --- | --- | --- |
| *csrA* | csrA2 | 5’- AAGAGAAATTTTGAGGGTGCGTCTCACCGATAAAGATGAGACGCGGAAAGATTACATATGAATATCCTCCTTAGTTCCTA-3’ | Datsenko mutation |  |
| *csrA* | csrA1-51 | 5’-gtaaatgccccgaaggaagtttctgttcaccgtg aagagatctaccagcgtGTGTAGGCTGGAGCTGCTT-3’ | Datsenko mutation |  |
| *csrB* | EH 12 | 5’-AACCAGCCATTCATCCTGATAG-3’ | Mutation transfer& validation | 1790 |
| *csrB* | EH 13 CsrBantisens | 5’-ATCTGATTGGTCATCTGGTGAC-3’ |
| *csrC* | EH 10 | 5’-TCAGCGCGCTGGATTTACCTGC-3’ | Mutation transfer& validation | 2253 |
| *csrC* | EH 11 CsrCantisens | 5’-TCTTCGGCACTCAGGGTTTCGC -3’ |
| *csrD* | csrDFD | 5’-AGCGCGCATTATTCTACGTGAAAACGGATTAAACGGCAGGTGTAGGCTGGAGCTGCTTC-3’ | Datsenko mutation | 1273 |
| *csrD* | csrDRV | 5’-GTATGCCCGCTTCCTCACTATCGGAGTTAACAC AAGGCCATATGAATATCCTCCTTAGTT-3’ |
| *csrA* | csrA+ | 5’-CAATGCCGTGGACTCGCTTCAC-3’ | Cloning ORFinto pGEM-T | 542 |
| *csrA* | csrA- | 5’-GGAGGTCTGACAGATAGTAGTAATGC-3’ |
| *csrA* | csrA3 | 5’-GTGTATATCGGCTAAACTTAGG-3’ | Sequencing of *csrA* mutation | 1990 |
| *csrA* | csrA4 | 5’-CTTCACGCTCAATTAGTCTG-3’ |
