# Supplementary material for: The Carbon Storage Regulator (Csr) System Exerts a Nutrient-Specific Control over Central Metabolism in Escherichia coli Strain Nissle 1917
Source: PLoS One. 2013 Jun 20;8(6):e66386. doi: 10.1371/journal.pone.0066386 (PMC3688793; doi:10.1371/journal.pone.0066386)
Supplement: Table S2 — Conversion factors between cell dry weight and optical density (OD600) for the wild-type E. coli Nissle 1917, ΔcsrBC and ΔcsrA51 mutants. (DOC) [file pone.0066386.s002.doc]

**Table S2.** Conversion factors between cell dry weights and optical density for the wild-type *E. coli* Nissle 1917, Δ*csr*BC and Δ*csrA*51 mutants in exponential growth on glucose and gluconate.

| **Strain** | **CDW conversion factor** (g_CDW_/OD_600_ unit) |
| --- | --- |
| **Nissle 1917 wild-type** | 0.40 ± 0.02 |
| **Nissle 1917 Δ*csr*BC** | 0.40 ± 0.03 |
| **Nissle 1917 Δ*csr*A51** | 0.47 ± 0.01 |
