# Supplementary material for: The Carbon Storage Regulator (Csr) System Exerts a Nutrient-Specific Control over Central Metabolism in Escherichia coli Strain Nissle 1917
Source: PLoS One. 2013 Jun 20;8(6):e66386. doi: 10.1371/journal.pone.0066386 (PMC3688793; doi:10.1371/journal.pone.0066386)
Supplement: Table S3 — Metabolite concentrations in the wild-type E. coli Nissle 1917, ΔcsrBC and ΔcsrA51 mutants. Statistical differences between wild-type and ΔcsrA51 mutant are given. (DOC) [file pone.0066386.s003.doc]

**Table S3.** Metabolite concentrations in the wild-type *Escherichia coli* Nissle 1917, Δ*csr*BC and Δ*csrA*51 mutants upon exponential growth on glucose and gluconate.

|  | Glucose | | | | | Gluconate | | | | |
| --- | --- | --- | --- | --- | --- | --- | --- | --- | --- | --- |
|  | Concentration (µmol/g CDW) | | |  |  | Concentration (µmol/ g CDW) | | |  |  |
|  | wild-type | Δ*csrBC* | Δ*csrA*51 | ρ-value^a^ | ratio | wild-type | Δ*csrBC* | Δ*csrA*51 | ρ-value^a^ | ratio |
| 1,3-diPG^b^ | 2.28± 1.06 | 3.89±3.76 | 1.90±0.15 | 0.350 | 0.83 | 4.45±0.56 | 3.30±3.00 | 3.68±0.73 | 0.110 | 0.80 |
| 2/3-PG | 1.42±0.11 | 1.67±0.53 | 1.12±0.23 | 0.075 | 0.80 | 4.20±0.33 | 4.66±0.45 | 2.66±0.20 | 0.002 | 0.65 |
| 6PG | 0.38±0.09 | 0.38±0.07 | 0.77±0.21 | 0.035 | 2.05 | 3.59±0.21 | 2.33±0.55 | 0.65±0.79 | 0.010 | 0.20 |
| Aco | 0.21±0.07 | 0.11±0.16 | 0.00±0.01 | 0.075 | <0.005 | ND | ND | ND | ND | ND |
| ADP | 0.71±0.06 | 0.82±0.25 | 0.91±0.27 | 0.160 | 1.30 | 0.95±0.29 | 1.01±0.35 | 0.80±0.19 | 0.255 | 0.85 |
| AMP | 0.16±0.03 | 0.11±0.09 | 0.21±0.06 | 0.170 | 1.30 | 0.20±0.05 | 0.40±0.10 | 0.17±0.02 | 0.155 | 0.90 |
| ATP | 3.50±0.29 | 3.21±0.03 | 2.86±0.48 | 0.080 | 0.80 | 3.51±0.15 | 4.08±0.15 | 2.95±0.24 | 0.015 | 0.85 |
| cAMP | 0.01±0.02 | 0±0.01 | 0.01±0.01 | 0.350 | 0.25 | 0.01±0.01 | 0.01±0.01 | 0.01±0.04 | 0.295 | 40.5 |
| CDP | 0.07±0.02 | 0.08±0.04 | 0.09±0.01 | 0.250 | 1.25 | 0.19±0.05 | ND | 0.18±0.02 | 0.367 | 0.95 |
| Cit | 1.01±0.90 | 0.81±0.33 | 0.40±0.14 | 0.255 | 1.40 | 0.27±0.11 | 0.35±0.25 | 0.57±1.05 | 0.335 | 2.15 |
| CMP | 0.10±0.04 | 0.13±0.04 | 0.13±0.01 | 0.235 | 1.30 | 10.46±4.15 | 10.57±2.59 | 6.75±3.40 | 0.150 | 0.65 |
| CTP | 0.83±0.02 | 0.77±0.07 | 0.76±0.12 | 0.220 | 0.90 | 0.76±0.16 | 0.91±0.06 | 0.65±0.05 | 0.175 | 0.85 |
| dADP^b^ | 1.36±0.50 | 2.34±2.20 | 2.22±0.25 | 0.110 | 1.65 | 2.52±0.65 | 3.03±1.29 | 1.91±0.42 | 0.125 | 0.75 |
| dATP^b^ | 8.13±0.64 | 7.92±0.66 | 7.16±1.45 | 0.190 | 0.90 | 8.94±0.57 | 11.40±1.14 | 5.41±1.13 | 0.010 | 0.60 |
| dCDP^b^ | 1.32±0.10 | 1.63±1.05 | 1.28±0.15 | 0.375 | 0.95 | 1.40±0.31 | 1.56±0.44 | 1.86±0.09 | 0.060 | 1.30 |
| dGTP^b^ | 3.16±4.48 | 1.27±4.21 | 1.13±0.15 | 0.320 | 0.35 | ND | ND | ND | ND | ND |
| dTDP^b^ | 2.30±0.63 | 2.66±1.41 | 1.69±0.30 | 0.195 | 0.75 | 2.05±0.19 | 0.25±0.07 | 1.45±0.55 | 0.095 | 0.70 |
| dTTP^b^ | 12.44±2.42 | 10.90±1.40 | 11.06±1.51 | 0.280 | 0.90 | 12.86±1.30 | 13.52±0.40 | 9.53±2.40 | 0.060 | 0.75 |
| F1P | 0.030±0.01 | 0.035±0.01 | 0.02±0.01 | 0.330 | 0.80 | 0.19±0.11 | 0.21±0.06 | 0.14±0.05 | 0.270 | 0.75 |
| F6P | 0.44±0.03 | 0.41±0.03 | 0.90±0.09 | 0.002 | 2.05 | 0.26±0.03 | 0.19±0.03 | 0.19±0.02 | 0.015 | 0.75 |
| FBP | 2.90±0.55 | 2.30±0.12 | 1.44±0.32 | 0.050 | 0.50 | 2.95±0.48 | 2.51±0.15 | 1.82±0.09 | 0.025 | 0.60 |
| Fum | 0.59±0.34 | 0.56±0.16 | 0.44±0.09 | 0.315 | 0.75 | 0.75±0.27 | 0.72±0.10 | 0.63±0.15 | 0.260 | 0.85 |
| G6P | 1.78±0.19 | 1.93±0.61 | 2.00±0.40 | 0.240 | 1.10 | 0.97±0.71 | 0.48±0.06 | 0.39±0.06 | 0.145 | 0.40 |
| GDP | 0.46±0.03 | 0.19±0.55 | 0.44±0.05 | 0.300 | 0.95 | 0.34±0.09 | 0.42±0.12 | 0.30±0.06 | 0.260 | 0.85 |
| GMP | 0.001±0.05 | 0.01±0.05 | 0.01±0.06 | 0.445 | 6.85 | 0.01±0.05 | 0.01±0.05 | 0.01±0.05 | 0.450 | 0.85 |
| M6P | 0.26±0.03 | 0.26±0.04 | 0.56±0.17 | 0.045 | 2.15 | 0.05±0.03 | 0.12±0.02 | 0.15±0.01 | 0.010 | 5.05 |
| Mal | 1.82±0.45 | 0.93±0.30 | 0.95±0.30 | 0.085 | 0.50 | 1.64±0.57 | 2.29±0.30 | 2.35±0.67 | 0.115 | 1.45 |
| PEP | 0.19±0.05 | 0.21±0.08 | 0.21±0.05 | 0.380 | 1.10 | 0.49±0.05 | 0.54±0.13 | 0.49±0.11 | 0.475 | 1.00 |
| PRPP | 1.31±0.42 | 1.33±0.71 | 0.64±0.14 | 0.125 | 0.50 | 1.16±0.21 | 2.15±0.42 | 0.95±0.05 | 0.105 | 0.80 |
| R1P | 0.01±0.01 | 0.01±0.01 | 0.01±0.01 | 0.405 | 0.85 | 7.81±0.81 | 42.15±9.24 | 17.85±4.34 | 0.025 | 2.50 |
| R5P | 0.75±0.06 | 0.73±0.28 | 0.71±0.16 | 0.320 | 0.95 | 1.93±0.45 | 2.60±0.16 | 3.38±0.42 | <0.005 | 1.75 |
| S7P^b^ | 4.63±0.32 | 4.17±1.19 | 12.8±1.45 | 0.015 | 2.75 | 19.87±1.49 | 27.75±2.53 | 52.88±2.65 | <0.005 | 2.65 |
| Suc | 4.44±0.28 | 5.34±1.52 | 1.98±0.46 | 0.002 | 0.45 | 3.34±0.39 | 5.58±0.85 | 6.69±0.56 | <0.005 | 2.00 |
| Tre6P | 0.01±1.16 | 0±1.10 | 0.44±0.34 | 0.345 | 46 | ND | ND | ND | ND | ND |
| UDP | 0.16±0.04 | 0.17±0.12 | 0.15±0.04 | 0.335 | 1.00 | 0.01±0.01 | 0.01±0.05 | 0.01±0.01 | 0.035 | 1.00 |
| UDP-Glc | 4.65±0.14 | 4.86±0.47 | 5.2±0.48 | 0.085 | 1.10 | 2.86±0.32 | 3.79±0.58 | 2.14±0.57 | 0.050 | 0.75 |
| UMP | 1.76±0.28 | 1.89±0.56 | 2.03±0.33 | 0.203 | 1.15 | 1.03±0.12 | 0.87±0.01 | 0.80±0.10 | 0.025 | 0.75 |
| UTP | 0.96±0.06 | 0.92±0.02 | 0.73±0.13 | 0.040 | 0.75 | 1.06±0.08 | 1.19±0.30 | 1.05±0.10 | 0.490 | 1.00 |

a Statistical differences between wild-type and Δ*csrA51* mutant samples were calculated using two-tailed Student’s T test assuming equal variance.

b For these metabolites only the ^12^C/^13^C ratio is given. This ratio represents the concentration of the metabolite in samples relative to the IDMS standard, which added at the same amounts in all samples.

Statistical differences between wild-type and Δ*csrA*51 mutant (calculated from three independent biological replicates) are given
