# Supplementary material for: The Carbon Storage Regulator (Csr) System Exerts a Nutrient-Specific Control over Central Metabolism in Escherichia coli Strain Nissle 1917
Source: PLoS One. 2013 Jun 20;8(6):e66386. doi: 10.1371/journal.pone.0066386 (PMC3688793; doi:10.1371/journal.pone.0066386)
Supplement: Table S4 — Adenylate energy charge. (DOC) [file pone.0066386.s004.doc]

**Table S4.** Adenylate energy charge in the wild-type *Escherichia coli* Nissle 1917, Δ*csr*BC and Δ*csrA*51 mutants upon exponential growth on glucose and gluconate.

| **Strain** | **Adenylate Energy Charge** | |
| --- | --- | --- |
|  | Glucose | Gluconate |
| **Nissle 1917 wild-type** | 0.88 ± 0.02 | 0.86 ± 0.02 |
| **Nissle 1917 Δ*csr*BC** | 0.88 ± 0.03 | 0.84 ± 0.03 |
| **Nissle 1917 Δ*csr*A51** | 0.83 ± 0.03 | 0.85 ± 0.02 |
